# Numerical Calculations of Electric Response Properties Using the Bubbles and Cube Framework

**DOI:** 10.1021/acs.jpca.5c00849

**Published:** 2025-04-02

**Authors:** Eelis Solala, Wen-Hua Xu, Pauli Parkkinen, Dage Sundholm

**Affiliations:** † Department of Chemistry, University of Helsinki, P.O. Box 55 (A.I. Virtanens plats 1), FI-00014 Helsinki, Finland; ‡ Key Laboratory of Synthetic and Natural Functional Molecule of Ministry of Education, College of Chemistry and Materials Science, Northwest University, Xi’an 710127, China

## Abstract

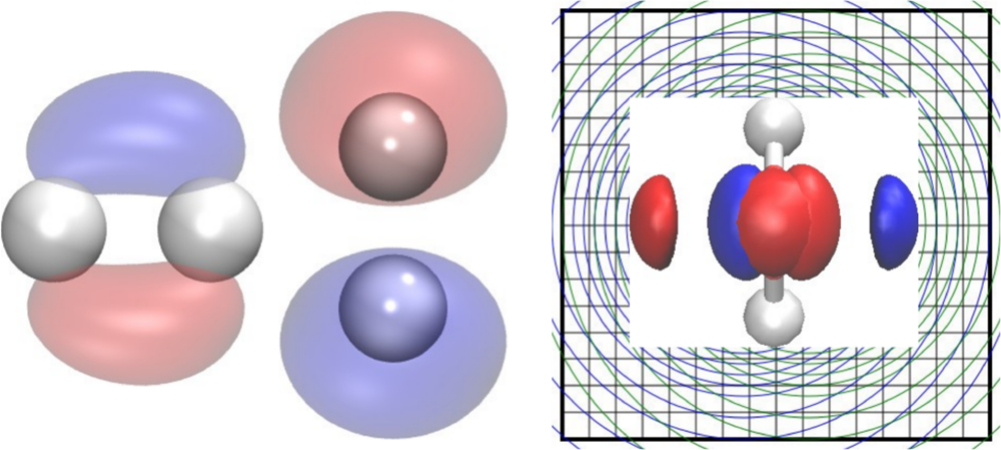

We have developed a fully numerical method for calculating the response of the Hartree–Fock orbitals to an external electric field. The Hartree–Fock orbitals are optimized using Green’s function methods by iterative numerical integration of the convolution with the Helmholtz kernel. The orbital response is obtained analogously by iterative numerical integration of the convolution with the Helmholtz kernel of the Sternheimer equation. The orbitals are expanded in atom-centered functions (bubbles), consisting of numerical radial functions multiplied by spherical harmonics. The remainder, i.e., the difference between the bubble expansion and the exact orbitals, is expanded in numerical tensorial local basis functions on a three-dimensional grid (cube). The methods have been tested by calculating polarizabilities for He, H_2_, and NH_3_, which are compared to the literature values.

## 1 Introduction

The paper “*Linear and nonlinear response functions for an exact state and for an MCSCF state*” by Jeppe Olsen and Poul Jo̷rgensen was the beginning of modern analytical response theory that is used for calculating a variety of time-dependent and time-independent second- and higher-order molecular properties using linear, quadratic, and cubic response functions.^1^ They developed the response theory for exact wave functions and showed how response theory can be efficiently employed in studies at multiconfiguration self-consistent field (MCSCF) levels of theory, which was at that time the state-of-the-art ab initio electron correlation level of theory. The paper has been cited about 1000 times because it is the starting point in the derivation and implementation of response theory at many levels of theory. Modern response approaches based on the article by Olsen and Jo̷rgensen are discussed in a comprehensive book by Norman et al.^2^

In this work, we have developed a fully numerical method for solving linear response equations by extending our bubbles and cube approach. We demonstrate this approach by calculating the polarizability of small molecules at the Hartree–Fock (HF) level of theory. The calculations are performed in the limit of complete basis sets. The complete basis-set limit is reached by expanding orbitals, potentials, and various auxiliary functions in a dual basis consisting of one-center functions at the nuclei and in a numerical basis on a three-dimensional (3D) equidistant Cartesian grid (cube), which is divided into elements of equal size. In each element, the 3D functions are expanded on a local basis consisting of the outer product of sixth-order Lagrange interpolation polynomials in the three Cartesian directions.

The one-center functions (bubbles) are expressed using radial functions multiplied with spherical harmonics.^3−8^ The radial part of the one-center functions is divided into elements that are shorter near the nucleus and longer farther away. Each element is divided into an equidistant grid. The functions in each element are expanded in sixth-order Lagrange interpolation polynomials.^5^ Similar approaches have also been suggested by other groups.^9,10^

There are alternative ways to handle the steep cusps in the vicinity of the atomic nuclei in fully numerical electronic structure calculations. A denser grid can be used near the nuclei^11−16^ or the steep part of the functions be eliminated by replacing the core electrons with soft pseudopotentials.^17,18^ Special coordinate systems can be used to distribute the grid points in numerical electronic structure methods for atoms and diatomic molecules.^19−24^ More references to numerical electronic structure approaches can be found in a recent review article.^25^ Response equations have also been solved in the basis-set limit by using a multiwavelet adaptive basis representation.^15,26−28^

In our approach, the bubble functions are obtained by projection, and the cube is expanded on a 3D grid. The division into bubbles and cubes is formally exact because what is not included in the bubbles is considered in the cube. The memory requirement due to the storage of the expansion coefficients of the cubes can be significantly reduced by using tensor decomposition methods.^29^

Fully numerical electronic structure methods are well aimed for massively parallel computers due to the real-space structure of the data. Computationally expensive calculations can be split into independent tasks when the spatial domain is divided into nonoverlapping regions, rendering grid-based fast multipole methods (GBFMMs) feasible.^3,4^ In real-space calculations, the data are easily organized when the values of the functions in the grid points are also the expansion coefficients of the orbitals, potentials, and auxiliary functions. Efficient algorithms can be designed by using prescreening and by introducing accurate approximations that speed up the calculations. The computational efficiency of fully numerical calculations of molecular properties exceeds the one of Gaussian basis-set calculations when very large basis sets are used.^15,26,28,30^

Differential equations such as the Schrödinger equation and the Poisson equation can be replaced with the Helmholtz and Coulomb integral equations, respectively, which consider the appropriate boundary conditions. The computational costs for numerically integrating the convolution of the Helmholtz and Poisson kernels appear to be significantly higher than the ones for solving the corresponding differential equations.^11−13,31−38^ However, numerical integration can be parallelized and efficient algorithms can be employed when expanding the unknown functions in local tensorial basis functions.^3,4,6,39−41^

Most of the computational time is spent in calculations of the cube parts of the orbitals and the potentials. However, the long-range part of the two-body interactions of the convolution integral of the Poisson and Helmholtz kernels can be identified and calculated using grid-based multipole expansions.^3,4^ The use of tensorial local basis functions implies that the short-range contributions to the electrostatic potentials and in the orbital optimization can be obtained by a series of matrix multiplications, which run efficiently on general-purpose graphics processing units (GPGPUs).^3,6^ The long-range contributions can be efficiently calculated using a GBFMM approach with octree partitioning of the spatial domain.^3^

Calculations of potentials showed that the computational wall time can even become independent of the system size, i.e., reaching an *N*^0^ scaling when the GBFMM approach is used in combination with a large number of GPGPUs.^3^ The same linear transformations are used when integrating the convolution with the Helmholtz kernel^4^ as used in calculations of the electrostatic potentials,^3^ implying that one can expect an *N*^0^ scaling also in the response calculations. The *N*^0^ scaling means that the computationally most expensive part of the calculation is faster than those parts of the calculation that are independent of the system size. However, the GBFMM algorithm and GPGPUs are not employed in the response calculations since the main aim of this work is to present the computational approach and to demonstrate its feasibility by a few examples.

The main part of this work was carried out in 2018. Completing the article has taken the time we needed to recover from the shock of Eelis Solala’s death due to sudden illness. He passed away on December 5, 2018, about one month before the planned submission of his doctoral thesis and this manuscript.

We organized the article into the following sections. In Section 2, we briefly present the bubbles and cube approach. Green’s function approach in Section 3 is used for calculating the electrostatic and exchange potentials as discussed in Section 3.1 as well as for optimizing the orbitals using numerical integration of the convolution of the Helmholtz kernel as described in Section 3.2. Solving the Fock equations using the bubbles and cube approach is outlined in Section 3.3. Green’s function approach for solving the response equations is described in Section 3.4. The accuracy of the implemented methods is demonstrated in Section 4 by calculating the polarizabilities for a few small molecules. The article is summarized in Section 5.

## 2 Bubbles and Cube Expansion

The scalar functions encountered in electronic structure calculations are often very steep in the vicinity of the nuclei. In order to accurately describe the behavior of these functions, many numerical electronic structure approaches have been proposed.^13,17,28,34,35,42−57^

In our bubbles and cube approach,^4−8^ the unknown functions *f*(**r**) are expanded in a double basis set consisting of atom-centered 1D functions on a dense radial grid multiplied with spherical harmonics and a 3D equidistant grid. The atom-center *f*^*A*^(*r*_*A*_, θ_*A*_, ϕ_*A*_) functions are called bubbles, and the *f*^Δ^(**r**) functions on the 3D grid are the cube
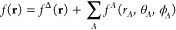
1

The angular part of the bubble functions is expanded in a number of spherical harmonics
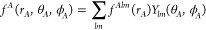
2and the one-dimensional (1D) radial functions *f*^*Alm*^(*r*_*A*_) are expanded in Lagrange interpolating polynomials (χ_*i*_(*r*_*A*_))
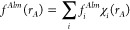
3

The radial range is divided into a number of elements, whose length is shorter closer to the nucleus and longer farther away from it. The grid points in each element are equidistant. The cube part of the functions is divided into equidistant ranges in the three dimensions, which are expanded in products of Lagrange interpolating polynomials (χ) on the grid
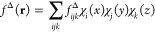
4

## 3 Green’s Functions

In electronic structure calculations, one can solve equations of the type
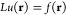
5where *L* is a linear operator, *f*(**r**) is a known function, and *u*(**r**) is the unknown function that one would like to know. One way to solve such equations is to construct the inverse of *L* operating on *f*(**r**) by using an integral expression
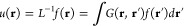
6where the kernel inside the integral is Green’s function of the operator *L*, which is defined as
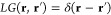
7in the physicist’s sign convention, where δ is Dirac’s delta function.

### 3.1 Poisson's Equation

The Poisson equation yielding the electrostatic interaction potential *V*(**r**) caused by a charge density ρ(**r**) is
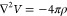
8

It can be reformulated and solved using Green’s function *G*_P_(**r**, **r′**), which is the Poisson kernel or Coulomb’s law for the electrostatic potential
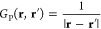
9The singularity can be circumvented by writing the Poisson kernel as an integral over an auxiliary dimension *t*
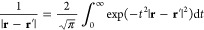
10

The integrand in eq 10 is separable in Cartesian coordinates
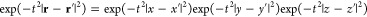
11which can be exploited when developing efficient algorithms. An alternative way to write the Poisson kernel is to use the Laplace expansion^58^
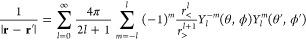
12where *r*_>_ = max(*r*, *r′*), *r*_<_ = min(*r*, *r′*), and *Y*_*l*_^*m*^ are the spherical harmonic functions.

Assuming that a charge density ρ(**r**) is totally confined inside a sphere of radius *R*, eq 12 is the multipole expansion of the electrostatic potential *V*(**r**) of the charge density outside it (|**r**| > *R*)
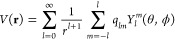
13where *q*_*lm*_ are the multipole moments
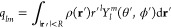
14

In practice, the multipole expansion is truncated at some finite value, *l*_max_, which enables a compression of the details of the charge distribution to a finite number of potential parameters.

### 3.2 Helmholtz Equation

The bound-state Helmholtz equation is
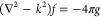
15where *k*^2^ > 0 is a constant. Green’s function is then given by
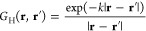
16

The potential obtained by integrating the convolution with this kernel is called the Yukawa potential,^59^ the screened Poisson potential, or the Debye–Hückel potential.^60^ The Helmholtz kernel has an integral expression similar to that of eq 10 for the Poisson kernel^61^
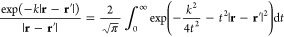
17

The Helmholtz kernel can also be written as a series expansion using complex spherical harmonics *Y*_*l*_^*m*^(θ, φ)^62^
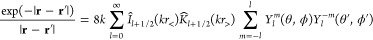
18where  and  are the modified spherical Bessel functions of order *l*. Functions obtained by convolution with the Helmholtz kernel can also be expanded in a multipole series similar to the electrostatic potential in the case of the Poisson kernel.^4,63−65^

### 3.3 Fock's Equation

The orbitals are optimized by iterative numerical integration of the convolution with the Helmholtz kernel instead of diagonalizing the Fock matrix, as in traditional self-consistent field (SCF) calculations. The integration of the convolution with the Helmholtz kernel *G*(**r**, **r***′*)*f*(**r***′*) is a linear operation implying that it can be performed separately for the bubbles and the cube parts
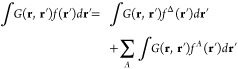
19where *f*^Δ^(**r***′*) is a smooth function that is expanded on the 3D grid, whereas the steep *f*^*A*^(**r***′*) functions in the vicinity of the nuclei are one-center functions. Problems originating from the singularity of the Helmholtz kernel in eq 17 are circumvented in the cube integration by introducing the integral transformation that depends on the orbital energy via .^4,7,8,13,35^

The *t* integral in eq 17 is calculated using the quadrature from *t* = 0 to *t*_*f*_, which is a large *t* value.
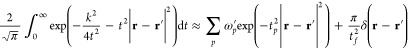
20

The integration in the last term in eq 20 is performed analytically from *t*_*f*_ to infinity. The *t*-integration weights ω_*p*_*′* of the convolution of the Helmholtz kernel depend on the orbital energy via *k* as
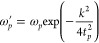
21where ω_*p*_ are the integration weights of the convolution with the Poisson kernel. *t*_*p*_ are *t*-integration points. The *t*-integration domain is divided into a linear region [0, *t*_*l*_], which is integrated using the Gaussian quadrature, the [*t*_*l*_, *t*_*f*_] interval is integrated using the Gaussian quadrature in logarithmic coordinates, and the integration in the interval of [*t*_*f*_, *∞*[ is calculated analytically. We used the same *t*-integration grid for integrating the convolution with the Helmholtz and Poisson kernels.

### 3.4 Response Equations

The electric polarizability tensor α is the first derivative of the dipole moment and the second derivative of the electronic energy with respect to the strength of the external electric field in the three Cartesian directions (ϵ, τ ∈ *x*, *y*, and *z*)
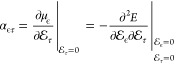
22

Polarizabilities can be obtained by calculating the total energy for a number of field strengths and differentiating  numerically at . Alternatively, the response formalism can be used.

In the presence of an external perturbation whose strength is λ, the Fock equation can be written as
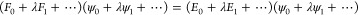
23where *F*_0_ is the unperturbed Fock operator, *E*_0_ is the unperturbed energy, and ψ_0_ is the unperturbed wave function. *F*_1_ is the first-order perturbed Fock operator, *E*_1_ is the first-order energy correction, and ψ_1_ is the first-order response of the wave function due to the perturbation. Considering contributions to the first order yields the modified Sternheimer equation^15,66,67^
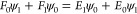
24

At the HF level, the first-order change in the density matrix can then be written as
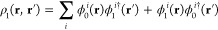
25where subscripts denote the order of the perturbation and summation runs over the occupied orbitals *i*. The perturbed Fock operator is
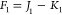
26where *J*_1_ is the perturbed Coulomb operator
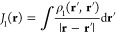
27and *K*_1_ is the perturbed exchange operator
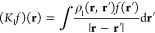
28

The idempotency condition of the density matrix leads to the weak orthogonality condition of the orbital response for occupied orbitals *i* and *j*
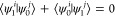
29of which the strong orthogonality condition ⟨ψ_0_^*i*^|ψ_1_^*j*^⟩ = 0 is a special case.

The modified Sternheimer equation in eq 24 can be written as a Helmholtz equation
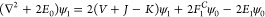
30where ∇^2^ originates from the kinetic energy operator, *V* is the nuclear attraction potential, *J* is the Coulomb repulsion potential between the electrons, and *K* is the exchange potential. In the presence of an external electric field in the  direction, the perturbed Fock operator is
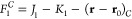
31

Introducing the orthogonality condition, one obtains
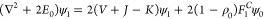
32where ρ_0_ is the unperturbed density matrix. The orbital response can be obtained by integrating the convolution with the Helmholtz kernel of the Sternheimer equation in the same way as that done when solving the Fock equation. Since the expression for the orbital response has terms that depend on the orbital response on its right-hand side, it must be solved iteratively. The orbital response is expanded in bubbles and cubes to avoid the numerical integration of steep functions. When the unperturbed orbitals and the orbital response are known, the perturbed density matrix and the polarizability tensor can be calculated as
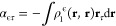
33where ϵ, τ ∈ *x*, *y*, *z* and
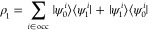
34

## 4 Results

The polarizability α_*zz*_ of the He atom was calculated at the HF level using a cubic grid whose sides are 6.5 bohr. The obtained α_*zz*_ values of 1.322233785 and 1.322233787 au are practically identical when using grids with step lengths of 0.10 and 0.05 bohr, respectively. The number of grid points is then 67^3^ = 300,763 and 133^3^ = 2,352,637, respectively. The α_*zz*_ values are in excellent agreement with the reference value of 1.32223373 au.^68^

Table 1 shows how the accuracy of the parallel and perpendicular components of the polarizability tensor of H_2_ as well as its trace is improved when increasing the length of the bubble expansion. The accuracy of α_⊥_ exceeds by 3 orders of magnitude the one obtained with large augmented correlation consistent basis sets. In the cube part, we used an equidistant 3D grid with 133 grid points in each Cartesian direction corresponding to a step length of about 0.1 bohr. The convergence criterion of the energy was 10^–9^ hartree. The calculations also show the importance of the *f*-type functions in the bubbles when using a small cube grid. The importance of the cube part diminishes when a more accurate bubble basis is used. One could, in principle, manage without the cube part when one is not aiming at calculations in the complete basis-set limit but at calculations that are more accurate than basis-set calculations using large Gaussian-type basis sets.

**Table 1:** Parallel and Perpendicular Components (in a.u.) of the Polarizability Tensor of H_2_ (*R* = 1.40028 bohr) as well as Its Trace^a^

*l*_max_	box size	α_∥_	α_⊥_	Tr (α)
2	12.5	6.39140	4.59423	5.19329
3	12.5	6.45030	4.61134	5.22433
4	12.5	6.45092	4.61144	5.22460
5	12.5	6.45114	4.61147	5.22469
MRMW^b^		6.452	4.612	5.225
aug-cc-pV5Z^c^		6.45086	4.60381	5.21950
aug-cc-pV6Z^c^		6.45140	4.60373	5.21962

a The polarizability was calculated at the HF level for different lengths (*l*_max_) of the bubble expansion. The spatial domain is 12.5 bohr in each cartesian direction. The obtained values are compared to polarizability tensors calculated using the multiresolution multiwavelet (MRMW) approach.^26^

b Ref (26).

c Calculated with Turbomole using the aug-cc-pV5Z and aug-cc-pV6Z basis sets.^69−73^

The elements of the polarizability tensor of NH_3_ calculated using different lengths (*l*_max_) of the bubble expansion are shown in Table 2. The number of grid points of the cube part is 133^3^, corresponding to a step length of about 0.1 bohr when the spatial domain is 12.5 bohr in each Cartesian direction. The molecular structure of NH_3_ belongs to the *C*_3*v*_ point group with an NH distance of 1.0120 Å, an HNH angle of 106.70°, and a torsion angle of 113.78°, which was also used in ref (26). The calculated polarizability tensor agrees well with the one calculated using the MRMW approach. The elements of the polarizability tensor calculated using very large augmented correlation consistent basis sets (5Z and 6Z)^72,73^ are also close to the ones obtained in the fully numerical calculations. The deviations appear in the third decimal.

**Table 2:** Elements of the Polarizability Tensor of NH_3_ Calculated at the HF Level Using Different Lengths (*l*_max_) of the Bubble Expansion^a^

*l*_max_	α_*xx*_	α_*yy*_	α_*zz*_	α_ave_
2	12.636	13.262	12.633	12.844
3	12.771	13.301	12.772	12.948
4	12.778	13.298	12.778	12.951
5	12.778	13.297	12.778	12.951
MRMW^b^	12.779	13.294	12.779	12.950
aug-cc-pV5Z^c^	12.773	13.275	12.773	12.940
aug-cc-pV6Z^c^	12.776	13.287	13.776	12.946

a The spatial domain is 12.5 bohr in each cartesian direction.

b Ref (26).

c Calculated with Turbomole using the aug-cc-pV5Z and aug-cc-pV6Z basis sets.^69−73^

## 5 Summary and Conclusions

We developed and implemented a method to numerically solve the Sternheimer equation. The orbital response is obtained by numerical integration of the convolution with the Helmholtz kernel of the Sternheimer equation instead of iteratively solving the corresponding linear response equations.^1^ The approach is iterative because the orbital response is obtained by integrating the convolution with the Helmholtz kernel that depends on the orbital response. We use dual numerical basis sets consisting of atom-like basis functions at each nucleus and a 3D Cartesian grid. The details of our bubbles and cube approach are discussed in ref (5).

Our numerical approach has many appealing features. The results converge systematically toward the basis-set limit when increasing the number of grid points of the cube or by increasing the number of angular momentum functions in the bubbles part or both. The bubble part of the calculations is very fast because they are one-center calculations. However, the basis-set convergence of the bubbles part is expected to be similar to the one when using, for example, Gaussian-type basis sets. The cube functions are expanded in local tensorial basis functions, implying that the numerical integration of the cube functions consists of a series of independent matrix multiplications that run efficiently on GPGPUs.^6^ The Helmholtz and Poisson kernels are six-dimensional two-body functions whose long- and short-range contributions are easily identified. The long-range part of the convolution integration can be replaced by general multipole expansions, making the computations significantly faster.^3,4^ Integral transformation of the singular two-body operator and discretization of the auxiliary dimension introduce an index that can be explored in parallel computations. Since the solution of the Sternheimer equation is expressed as a convolution integral with the Helmholtz kernel, it can be made faster using the same GBFMM approach as used in the orbital optimization.

We have calculated the polarizability tensor for He, H_2_, and NH_3_ at the HF level. The obtained polarizabilities agree well with values previously obtained using the MRMW approach. Other linear response properties can be calculated analogously. We used equidistant grid points in each element. However, a higher accuracy with the same number of grid points would be obtained by using for example a Gauss–Lobatto grid instead of the equidistant grid.^24^
